# The influence of meal size on prey DNA detectability in piscivorous birds

**DOI:** 10.1111/1755-0998.12706

**Published:** 2017-10-14

**Authors:** Bettina Thalinger, Johannes Oehm, Armin Obwexer, Michael Traugott

**Affiliations:** ^1^ Institute of Ecology University of Innsbruck Innsbruck Austria

**Keywords:** cormorant, dietary sample, feeding experiment, molecular prey detection, molecular scatology, prey detection interval

## Abstract

Molecular methods allow noninvasive assessment of vertebrate predator–prey systems at high taxonomic resolution by examining dietary samples such as faeces and pellets. To facilitate the interpretation of field‐derived data, feeding trials, investigating the impacts of biological, methodological and environmental factors on prey DNA detection, have been conducted. The effect of meal size, however, has not yet been explicitly considered for vertebrate consumers. Moreover, different noninvasively obtained sample types remain to be compared in such experiments. Here, we present a feeding trial on abundant piscivorous birds, Great Cormorants (*Phalacrocorax carbo*), to assess meal size effects on postfeeding prey DNA detection success. Faeces and pellets were sampled twice a day after the feed of large (350–540 g), medium (190–345 g) and small (15–170 g) fish meals contributing either a large (>79%) or small (<38%) share to the daily consumption. Samples were examined for prey DNA and fish hard parts. Molecular analysis of faeces revealed that both large meal size and share had a significantly positive effect on prey DNA detection rate postfeeding. Furthermore, large meals were detectable for a significantly longer time span with a detection limit at ~76 hr and a 50% detection probability at ~32 hr postfeeding. In pellets, molecular methods reliably identified the meal consumed the previous day, which was not possible via morphological analysis or when examining individual faeces. The less reliable prey DNA detection of small meals or meal shares in faeces signifies the importance of large numbers of dietary samples to obtain reliable trophic data.

## 1 INTRODUCTION

Molecular methods are widely used to investigate trophic interactions, and the methodology itself has become almost as diverse as the ecosystems and interactions investigated (Symondson & Harwood, 2014). Biological, methodological and environmental factors affect prey DNA detection success, and for a meaningful interpretation of field‐derived molecular trophic data, it is indispensable to understand how these factors influence postfeeding prey detection over time (e.g., King, Read, Traugott, & Symondson, 2008; Pompanon et al., 2012; Traugott, Kamenova, Ruess, Seeber, & Plantegenest, 2013). Herein, meal size can play an important role and critically affect the detection of trophic interactions (Greenstone, Payton, Weber, & Simmons, 2014).

Invertebrate feeding trials show that meal size can have profound effects on postfeeding prey DNA detection intervals in gut content samples (Gagnon, Doyon, Heimpel, & Brodeur, 2011; Hagler & Blackmer, 2013). For example, King, Vaughan, Bell, Bohan, and Symondson (2010) have found that earthworm DNA can be detected significantly longer in carabid beetles’ guts after ad libitum consumption (50% detection probability at 80.3 hr postfeeding) compared to beetles which were only allowed restricted feeding (50% detection probability at 17 hr postfeeding). Even so, there are several studies involving similar predator taxa which do not find an effect of meal size on postfeeding prey DNA detection success: the consumption of one to six mosquitoes or 10‐fold variation in cockchafer larvae consumption had no detectable effect in experiments with carabid beetles (Juen & Traugott, 2005; Zaidi, Jaal, Hawkes, Hemingway, & Symondson, 1999).

Compared to invertebrate consumers, little is known about the effect of meal size on prey DNA detection success in vertebrates at different time points postfeeding. Molecular stomach content studies have been limited to fish feeding on fish eggs and larvae, and small mammals consuming frogs (e.g., Carreon‐Martinez, Johnson, Ludsin, & Heath, 2011; Egeter, Bishop, & Robertson, 2015; Hunter, Taylor, Fox, Maillard, & Taylor, 2012; Rosel & Kocher, 2002). Whilst meal size was kept constant by Egeter et al. (2015) and Hunter et al. (2012), as suggested for arthropod feeding trials (Greenstone et al., 2014), no effect of meal size was observed for small fish meals by Carreon‐Martinez et al. (2011). In vertebrate feeding trials and field studies, however, faeces and regurgitates are typically used as the source of prey DNA (e.g., Alonso et al., 2014; Deagle, Thomas, Shaffer, Trites, & Jarman, 2013). The utilization of these samples is based on the fact that a proportion of prey DNA remains detectable despite digestive processes in the stomach and intestine (Deagle et al., 2007; Egeter et al., 2015). This shifts the focus of experiments away from sheer presence/absence gut content examinations towards investigations of digestion rates. So far, faecal samples have been used to study gut transition times, that is, the intervals between first and last prey detection, of carrion crows feeding on cockchafer larvae as well as sea lions and seals consuming up to 10 different prey species (Casper, Jarman, Deagle, Gales, & Hindell, 2007; Deagle et al., 2005; Oehm, Juen, Nagiller, Neuhauser, & Traugott, 2011). Several studies used fixed shares of prey species in the diet of vertebrates to assess the effect of differences in meal share on prey detectability (Casper et al., 2007; Deagle, Chiaradia, McInnes, & Jarman, 2010; Deagle et al., 2005; Peters, Ophelkeller, Bott, & Goldsworthy, 2015). Additionally, molecular prey detection probability over time was compared between faeces and stomach samples of small mammals (Egeter et al., 2015), but we lack information on how meal size affects prey DNA detection success in such dietary samples.

Here, we address this gap of knowledge and investigate how meal size and the share of a meal in the daily consumption (i.e., depending on the total amount of fish consumed during 1 day, the per cent contribution to the daily consumption can vary considerably for individual meals) influence the postfeeding prey detection interval and the prey detection probability over time for a vertebrate predator–prey system. Faeces and pellets obtained in a feeding trial where cormorants fed on different fish species were used as dietary samples. The daily consumption depended on the total amount of fish consumed during 1 day and the per cent contribution of a particular fish species to the daily consumption varied considerably for individual meals. Cormorants and shags (Phalacrocoracidae) are abundant piscivores, foraging worldwide in coastal and inland waters of Europe, Asia, Africa and along the east coast of the United States (del Hoyo, Elliot, & Sargatal, 1992). Their trophic ecology has been primarily studied through morphological analysis of pellets, which are regurgitated every morning and contain the indigestible prey remains of the previous day (Zijlstra & van Eerden, 1995). However, consumed fish are not reliably detected via hard part analysis, and species‐specific morphological identification of prey remains is not always possible (Barrett et al., 2007). Hence, the use of molecular methods is advisable to detect all consumed prey species. The application of molecular methods furthermore permits the utilization of faeces, which do not usually contain easily identifiable prey hard parts (Johnson & Ross, 1996), and allows a re‐evaluation of gut transition times for cormorants, which have been previously examined with dye capsules hidden in fish (Brugger, 1993).

The current study involved four captive Great Cormorants (*Phalacrocorax carbo*) which were kept individually and subjected to a feeding regime with six fish species offered in large (350–540 g), medium (190–345 g) and small (15–170 g) quantities and in a fixed order. Depending on the food intake of the individual bird, different meals could either contribute a large (>79%) or small (<38%) share to the daily consumption. The faeces and pellets produced by the birds were collected twice a day and analysed molecularly and morphologically to test the following hypotheses: (H1) The use of molecular methods allows reliable detection of fish DNA in faeces and pellets from 2–4 hr to 48 hr postfeeding, based on the results of Brugger (1993). (H2) Prey DNA stemming from large meals or meals contributing a large share to the daily consumption has a significantly higher detection rate compared to small and medium meals as well as small shares. Additionally, large meals/shares should lead to significantly longer postfeeding prey DNA detection intervals than small and medium ones.

## 2 MATERIALS AND METHODS

In February 2014, a feeding trial on adult captive cormorants was permitted by and carried out at “Wilhelma The Zoological and Botanical Gardens” (Stuttgart, Germany). Three days prior to the start of the experiment, clipped cormorants were moved from their enclosure to one of the aviaries in which the feeding trial took place. They were kept together to reduce stress and were fed with *Rutilus rutilus* (common roach). At the start of the experiment, each bird was placed into one of neighbouring aviaries, each 9.1 m² in size, the floor covered with plastic foil, and containing a perch and a 65 × 45 × 20 cm plastic tub filled with water. A feather sample was taken from each bird for molecular sexing (Supporting information) resulting in the identification of two females and two males.

### 2.1 Fish feeding regime

**Figure 1:**
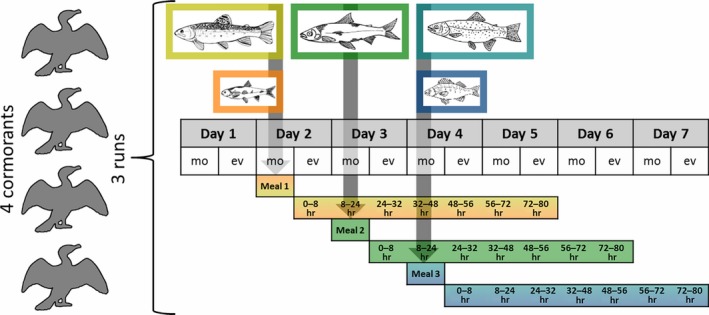
The set‐up of the feeding trial with four cormorants, each participating in three experimental runs, the consumption of the five fish meals (*Salvelinus fontinalis* and *Leuciscus leuciscus* on day 2, *Coregonus* spp. on day 3, *Oncorhynchus mykiss* and *Perca fluviatilis* on day 4), the samplings (mo: morning, ev: evening) and the seven detection time steps (0–8 hr … 72–80 hr) following each fish meal. The base diet of *Rutilus rutilus*, fed before and after, is not displayed

The feeding trial consisted of three consecutive experimental runs, each lasting 6 days and intermitted each time by 3 days of *R. rutilus* feeding. Each 6‐day run included 3 days on which the cormorants were provided with a different fish ration consisting of one or two meals (i.e., fish species) every morning at ~ 8:30 a.m. and 3 days of *R. rutilus* feeding. On day 1, the birds were offered *R. rutilus*, followed by a combination of *Salvelinus fontinalis* (brook trout) and *Leuciscus leuciscus* (common dace) on day 2, *Coregonus* spp. (whitefish) on day 3 and a combination of *Oncorhynchus mykiss* (rainbow trout) and *Perca fluviatilis* (European perch) on day 4, before switching back to *R. rutilus* for the last 2 days and the 3‐day break between experimental runs (Figure 1). *Salvelinus fontinalis, Coregonus *spp. and *O. mykiss* were offered in medium‐to‐large quantities (170–540 g), whilst only one small fish of *L. leuciscus* and *P. fluviatilis* (15–40 g) was included in the respective rations. All fish offered to the birds during the 6‐day runs were gutted to preclude potential influence of secondary predation, measured (total length), weighted, thoroughly washed and stored in plastic bags at ~ 4°C the previous evening using gloves and DNA‐free scalpels to avoid contamination. Each ration was offered to the cormorants in the water tub, and fish not consumed were removed in the evening. Throughout the day, the aviaries were checked every 2–3 hr to record fish consumption.

### 2.2 Sampling of faeces and pellets

Each morning (~ 8:30 a.m.; morning samplings mo1‐7) and early evening (~ 4:30 p.m., evening samplings ev1‐6) faecal samples were collected, starting right before the first feed of gutted *R. rutilus* and ending 7 days later in the morning. If possible, faeces of rather “solid” consistency were collected using gloves, reaction tubes, and in the case of fluid samples, DNA‐free plastic syringes. About five faeces were collected (min. one; max. ten) per bird and sampling and their colour noted. Regurgitated pellets were collected in plastic bags. To avoid contamination, the plastic foil was removed after each sampling and aviaries and cormorants were thoroughly cleaned with water before lining the aviaries with fresh foil. Finally, the water tub was washed with bleach (sodium hypochlorite, 3%) every day before providing the new fish meal. All work was carried out wearing gloves and without stepping onto fresh foil. The obtained samples were individually stored at – 32°C upon DNA extraction. In total, 855 faeces and 28 pellets were collected. Due to the exclusion of white faeces after initial tests for prey detectability (see below), only 706 faecal samples were used for the subsequent analyses. With one exception, pellets were collected during morning sampling.

### 2.3 Molecular prey detection

The molecular work was carried out in a special diagnostic molecular laboratory at the Institute of Ecology, University of Innsbruck (Austria). All samples were lysed with a mixture of TES‐buffer (0.1 m TRIS, 10 mm EDTA, 2% sodium dodecyl sulphate; pH 8) and Proteinase K (20 mg/ml) in a ratio of 190:1. The amount of lysis buffer depended on the size of the sample: 300 μl for small, 500 μl for medium, and 3 ml for large faeces with a volume below 100 μl, 100 – 500 μl and above 500 μl, respectively. Cormorant pellets were also separated into three size classes, and 3 ml lysis buffer was added to small, 5 ml to medium and 8 ml to large samples (cf. Thalinger et al., 2016). All samples were vortexed and incubated on a rocking platform overnight at 56°C; 1 ml of pellet lysate was transferred to a new reaction tube and set aside for DNA extraction, whilst the remainder of the sample was devoted to morphological analysis.

The BioSprint 96 DNA blood Kit (Qiagen) was used for DNA extraction on the BioSprint 96 instrument (Qiagen) in accordance with the manufacturer's instructions, but using TE‐buffer instead of AE‐buffer for DNA elution. Ninety‐two lysates and four extraction controls, containing solely TES‐buffer, were processed at once. The controls were checked for cross‐contamination with the FishTax multiplex PCR assay designed to detect all fish species used in this trial at a family‐specific level using the Multiplex PCR Kit (Qiagen) in 10 μl PCRs including 1.5 μl DNA extract, one‐time reaction mix, 5 μg BSA, 30 mm TMAC, primers in respective concentrations and PCR‐grade water; optimized thermocycling conditions were 15 min at 95°C, 35 cycles of 30 s at 94°C, 90 s at 64°C, 1 min at 72°C and 10 min at 72°C once (cf. Thalinger et al., 2016) and all extraction controls resulted negative. To test the suitability of white/grey faeces, putatively containing high levels of urea and little amounts of prey DNA, for molecular prey detection, DNA extracts of 623 brown and 83 white samples were tested with the FishTax multiplex PCR assay under the conditions described above (cf. Thalinger et al., 2016). The influence of potential PCR inhibitors such as ammonia and bile salts, included especially in white/grey faecal samples, was evaluated via a subset of faecal extracts (30 brown, 30 white/grey) testing negative in the FishTax multiplex PCR. These were spiked with ~50 ng *P. fluviatilis* DNA to test for PCR inhibition. Independent of faeces colour, all initially negative samples resulted positive in the spike PCR.

DNA extracts of faeces and regurgitated pellets were screened for fish DNA using singleplex PCRs with specific primers (partly taken from Thalinger et al. (2016) and partly new) for the six fish species included in the trial (Table 1). The 10 μl PCRs included 3.2 μl DNA extract, one‐time Multiplex PCR Kit (Qiagen) reaction mix, 5 μg BSA, 30 mm TMAC and 0.5 μm per forward and reverse primer. The optimized thermocycling conditions were 15 min at 95°C, 35 cycles of 30 s at 94°C, 90 s at 64°C or 66°C (*R. rutilus*), 1 min at 72°C and 10 min at 72°C once. For *L. leuciscus,* two reverse primers (binding at the same site; 0.25 μm each) were used simultaneously to account for a potential one‐base difference at the primer‐binding site. To minimize the effect of target gene copy number and amplicon length difference, all assays target 82‐ to 94‐bp fragments of the cytochrome *c* oxidase subunit I (COI) gene. The primer pairs were tested successfully for their specificity with DNA extracts of cormorant tissue and fish tissue of the other fish species fed during the trial. To determine PCR sensitivity for each primer pair, dilution series of standardized DNA templates of the target species were carried out in the presence of ~300 ng cormorant DNA (Sint, Raso, & Traugott, 2012). For all assays, 10 target DNA ds were sufficient to reliably produce amplicons with a signal strength above 0.1 relative fluorescence units (RFU; see below). Finally, 30 PCR products each testing positive for one of the fed fish species were sequenced; all of them confirmed the amplification of the target species and no amplification of cormorant or nontarget fish DNA. Thus, the primers listed in Table 1 are specific for their respective target species under the presented thermocycling conditions and do not amplify cormorant DNA or nontarget fish DNA. For all samples, the automated capillary electrophoresis system QIAxcel with the corresponding software qiaxcel screengel version 1.4.0 (Method AM320; Qiagen) was used for PCR product separation and analysis with DNA fragments of the expected length producing a signal ≥0.1 RFU being deemed positive.

**Table 1:** A summary of the singleplex PCR assays all targeting the cytochrome *c* oxidase subunit I (COI) gene

Target taxon	Primer name	Primer sequence (5′–3′)	Fragment length (bp)	Sensitivity (DNA ds)	Source	Primer concentration in PCR (μm)
*Rutilus rutilus*	Rut‐rut‐S665	TTCYGGTGTTGAGGCCGGT	94	10	Thalinger et al. (2016)	0.5
	Rut‐rut‐A665	TGTTAAATCTACTGATGCCCCG			Thalinger et al. (2016)	0.5
*Leuciscus leuciscus*/*idus*	Leu‐lid‐S663	CATCTCCCAGTATCAAACACCG	87–88	10	Thalinger et al. (2016)	0.5
	Leu‐lid‐A1008	CCGGCAGCTAAGACTGGTAAT				0.25
	Leu‐lid‐A1009	CGGCAGCTAAGACTGGCAAT				0.25
*Oncorhynchus mykiss*	Onc‐myk‐S655	TCTCCCTTCATTTAGCTGGAATC	82	10	Thalinger et al. (2016)	0.5
	Onc‐myk‐S655	GCTGGAGGTTTTATGTTAATAATGGTC			Thalinger et al. (2016)	0.5
*Salvelinus* spp.	Sal‐vel‐S1007	GCCTCTTAATTCGGGCAGAGT	93	10		0.5
	Sal‐vel‐A651	TAACGAAGGCATGGGCTGTT			Thalinger et al. (2016)	0.5
*Coregonus* spp.	Cor‐spp‐S1006	GCCGTCTTAATTACCGCAGTG	84	10		0.5
	Cor‐spp‐A1006	ATTCCGGTCTGTGAGTAGCATG				0.5
*Perca fluviatilis*	Per‐flu‐S1005	TGCTTCTCACAGACCGAAACTTG	82	10		0.5
	Per‐flu‐A1005	AATAAGTGTTGGTAAAGAATAGGGTCG				0.5

The target taxon of each primer combination, primer names and sequences, their corresponding amplicon sizes, primer concentration in PCR and the sensitivity of each singleplex PCR in DNA double‐strands (ds) necessary to reliably detect a target taxon in a sample with mixed target and nontarget DNA are provided. Additionally, the source column states whether a primer has been previously published.

### 2.4 Morphological analysis

Pellet lysates were sieved (0.5‐mm mesh size) and rinsed with water to remove soft parts and mucosa. Otoliths, pharyngeal bones, chewing pads and jaws, which allow fish identification, were separated from other hard parts. The detected remains were identified using the identification keys of Harkonen (1986), as well as reference collections provided by Werner Suter (Swiss Federal Research Institute, Birmensdorf, Switzerland), Josef Trauttmansdorff (Otto König Institute, Stockerau, Austria) and the Bavarian State Collection of Zoology (Munich, Germany). The morphological analysis of faeces was omitted because they rarely contain hard parts (Johnson & Ross, 1996; Zijlstra & van Eerden, 1995) and no hard parts were found in a visually inspected subsample of 100 faeces.

### 2.5 Statistical analysis and model selection

Chi‐square calculations including Yates corrections were made to test whether white and brown faeces significantly differed in prey DNA detection success. White faeces were significantly less likely (χ² = 45.03, *p* < .001) to test positive in PCR compared to brown faeces; thus, the remaining 149 white coloured faeces were not DNA‐extracted. Whenever cormorants did not consume a whole meal (i.e., up to four individual fish) at once and feeding was not directly observed, the consumption interval was calculated and ranged from zero (direct observation; bird consumed the whole meal) to eight (bird consumed the first fish of a meal right after the ration was provided and the last one prior to evening sampling) hours. The mean of this time frame was then used to determine gut passage time.

Based on the actual fish consumed, meals of each fish species were assigned to three classes: “large” (350–540 g), “medium” (190–345 g) and “small” (15–170 g); each meal further contributed a “large” (>79%) or “small” (<38%) share to the total daily consumption. Sometimes cormorants did not consume the entire daily ration; thus, small meals in four of 26 cases represented a large share. For faeces, three types of prey detection variables were used for statistical analysis: (i) overall detection rates, (ii) fish meal detection rates; and (iii) detection as a binary variable. Overall detection rates were calculated for morning and evening sampling events of the 6‐day feeding regime by pooling the data obtained from all cormorants during the three experimental runs. The fish meal detection rates were calculated per sampling, bird and fish species (i.e., if two of four faeces collected during one specific sampling session in one aviary were positive for, for example, *S. fontinalis*, the respective fish meal detection rate equalled 0.5). Detection as a binary variable (yes/no) was reported for all analysed faeces and detection events for each prey species individually. *Rutilus rutilus,* used as a base diet, was excluded from all analyses except for overall detection rate calculations. The time postfeeding was initially not treated as a continuous variable due to the large sampling interval; instead, seven time steps (0–8, 8–24, 24–32, 32–48, 48–56, 56–72 and 72–80 hr), during which faeces could have been produced, were specified. For each time step, the relationship between meal size (small, medium and large) and meal share (small, large) was plotted against the fish meal detection rate.

We analysed fish meal detection rate (58 meals) using seven time steps (i.e., seven time points of detection per meal), three meal sizes, two meal shares, fish species, experimental run (1–3), cormorant (1–4), cormorant sex and sampling (morning, evening) as categorical predictor variables in logistic general linear models (Dobson, 1990; Papke & Wooldridge, 1996; Zao, Chen, & Schaffner, 2001). Due to the small number of cormorants and experimental runs, no models with random factors were considered (Bingham, 2004). For all variables except time step, the different categories were entered via dummy coding into the models using “large meal,” “large share,” “experimental run 1,” “cormorant 1,” “female” and “evening sampling” as base categories for comparison of different variable levels. As a decline in prey detection rate over time can be expected (Greenstone et al., 2014), forward difference coding was used to detect differences in fish meal detection rates between adjacent time steps (Collett, 2015). Based on the focus of this study to investigate the effects of meal size and meal share on fish meal detection rate over time, five models were chosen for comparison (Table 2). As no overdispersion was detected for the full model (ĉ < 1, but set to 1 for subsequent analyses (Burnham & Anderson, 2002)), model performance was evaluated based on AICc, ΔAICc and AICc weights (ω) as a metric for the strength of evidence supporting each of the five models as the best description of the data (Burnham & Anderson, 2002). Additionally, the Nagelkerkes/Cragg & Uhlers pseudo‐*R* ^2^ (Nagelkerke, 1991) was calculated for each of the candidate models as a measure of model performance compared to an intercept‐only null model (Burnham, Anderson, & Huyvaert, 2011). As only one of the candidate models had substantial weight (ΔAICc ≥ 7 for all other models), no model‐averaged parameter values were calculated.

**Table 2:** The set of candidate models (simplified formula) used to investigate the effects of time since consumption, meal size and meal share on fish meal detection rate (“p”)

Model #	Model description
1	$\text{ln}\left(\frac{p}{1-p}\right)=\text{time step}+\text{meal size}+\text{meal share}+\text{fish species}+\text{sampling}+\text{experimental run}+\text{cormorant}+\text{cormorant sex}$
2	$\text{ln}\left(\frac{p}{1-p}\right)=\text{time step}+\;\text{meal size}+\text{meal share}+\text{fish species}$
3	$\text{ln}\left(\frac{p}{1-p}\right)=\text{time step}+\text{meal size}+\text{meal share}$
4	$\text{ln}\left(\frac{p}{1-p}\right)=\text{time step}+\text{meal size}$
5	$\text{ln}\left(\frac{p}{1-p}\right)=\text{time step}$

All explanatory variables were categorical: time step (0–8 hr … 72–80 hr), meal size (large, medium and small) and share of a meal in the daily fish consumption (large and small), fish species (*Salvelinus fontinalis*,* Leuciscus leuciscus*,* Coregonus* spp., *Oncorhynchus mykiss* and *Perca fluviatilis*), sampling (morning and evening), experimental run (1, 2 and 3), cormorant (individual 1, 2, 3 and 4) and cormorant sex (female and male).

Finally, a data set containing detection as a binary variable and faeces collected on seven time steps post consumption was used to calculate the detection probability over time. This was computed separately for small (757 yes/no events), medium (665 yes/no events) and large meals (346 yes/no events) as well as large (1 124 yes/no events) and small meal shares (644 yes/no events) with PROBIT regressions treating time as continuous variable (cf. Greenstone et al., 2014), including their 95% confidence intervals (CIs) and Nagelkerkes/Cragg & Uhlers pseudo‐*R* ^2^. Whilst other studies (cf. Greenstone et al., 2014) use this kind of analysis to report the time at which prey is detectable in a certain share of individuals, here, detection probability over time is pooled across the faeces produced by four cormorants during three experimental runs. The calculations and visualizations were made with r (R Development Core Team 2015) and the packages aiccmodavg (Mazerolle, 2017), drc (Ritz & Streibig, 2005), gg2plot (Wickham, 2009), gridextra (Auguie, 2015) and mumin (Barton, 2016) as well as sigmaplot 13.0 (Systat Software, Inc., San Jose, USA).

## 3 RESULTS

**Figure 2:**
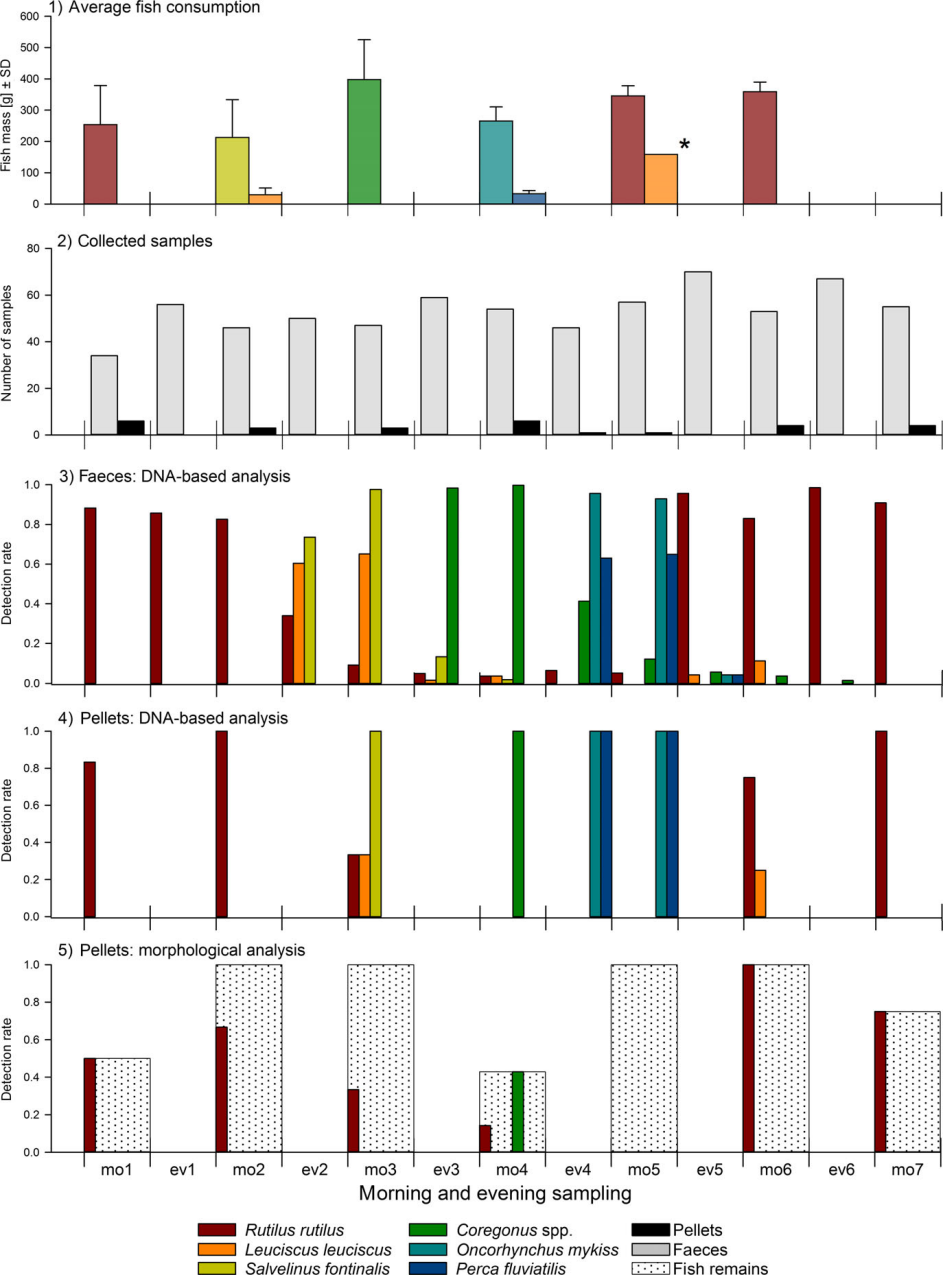
The average fish consumption per bird, number of collected faeces and pellets, and the overall detection rates within these sample types summed up over cormorants and experimental runs. On the *x*‐axis, mo1–7 and ev1–7 denote the morning and evening samplings, respectively, which were repeated three times during the whole trial. From top to bottom: panel 1: mean consumed fish mass per species based on 93 meals; panel 2: number of analysed faeces (*n* = 706) and pellets (*n* = 28) per sampling; panel 3: DNA‐based species‐specific fish detection rates in faeces; panel 4: DNA‐based species‐specific fish detection rates in pellets; panel 5: species‐specific fish detection rates obtained through morphological pellet analysis; white‐dotted background bars show proportion of pellets in which fish remains were found. The asterisk marks an accidentally fed large *Leuciscus leuciscus* meal

The mean consumed fish mass per day and bird throughout the experiment (3 × 6 days) was 311 g ± 118 g (*SD*), regardless of fish species (see panel 1, Figure 2 for mean consumed fish mass per species). In total, 98.5% of the offered fish mass was consumed and 34 to 70 faeces and one to six pellets were collected per sampling event (pooled across experimental runs and cormorants; panel 2, Figure 2). Regarding overall detection rates (i), species‐specific DNA‐based prey detection rates in faeces ranged from 99% to 1.5%. Species consumed in quantities below 40 g were detected in up to 60% (*L. leuciscus*) and 63% (*P. fluviatilis*), of the faeces collected at the first sampling session postfeeding. Medium and large meals were detected in over 74% of the faeces at this time point (panel 3, Figure 2). The earliest prey detections happened 1.5–2.5 hr after mean consumption, but fish consumption occurred in all these cases in a 2‐h window between check‐ups. The latest detection of prey DNA was ~75 hr postfeeding (*Coregonus *spp., 495 g, and 100% of daily ration). All consumed fish species were detected molecularly in pellets (panel 4, Figure 2). Using morphological analysis, fish remains were detected in 73% of the pellets; however, *R. rutilus* and *Coregonus* spp. were the only species that could be identified (panel 5, Figure 2).

**Figure 3:**
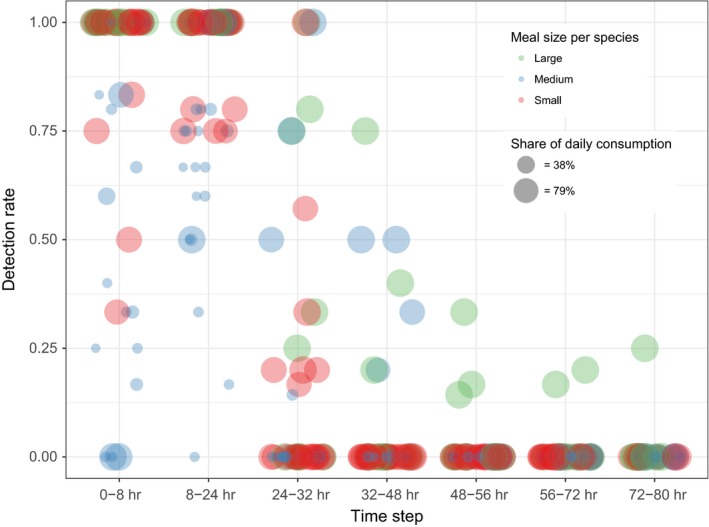
The relationship between meal size, share of the meal in the daily fish consumption and fish meal detection rate (per bird and sampling) based on 58 meals, 558 faeces and 376 detection rates distributed across seven time steps (0–8 hr … 72–80 hr). Large meals (350–540 g) are depicted by green circles, medium meals (190–345 g) by blue circles and small meals (15–170 g) by red circles. The share of each meal in the daily fish consumption is displayed as a continuous variable and coded by the size of the circles; for easy identification of small (<38%) and large (>79%) meal shares, the legend displays two circle sizes representing 38% and 79% shares

Fish meal detection rates (ii) of all large meals reached 100% at the time intervals 0–8 hr and 8–24 hr. Medium and small meals were detectable up to 24–32 hr and 32–48 hr, respectively; small shares were, with one exception, only detectable during the first two time steps (Figure 3). The ΔAICc‐based comparison of model weight led to the selection of one logistic generalized linear candidate model (model 3) for fish meal detection rate (ω = 0.96, pseudo‐*R* ^2^ = 0.87, AIC = 545.41; Table 3). It contained time step, meal size and meal share as explanatory variables, thus omitting the influences of fish species, experimental run, cormorant, cormorant sex and sampling (Table 3). Based on forward difference coding, fish meal detection rates did not differ significantly between 0–8 hr and 8–24 hr. From 8–24 hr to 48–56 hr, the detection rates were significantly lower at the latter time step compared to the previous one, whilst they did not differ significantly between adjacent time steps greater than 48–56 hr (Table 4). Compared to large meals, medium meals had significantly lower fish meal detection rates; this was also true for small shares compared to large shares (Table 4).

**Table 3:** Results of ordinal ranking based on ΔAICc for the logistic generalized linear models of fish meal detection rate via categorical predictor variables

Model #	*K*	AICc	ΔAICc	ω	*R* ^2^
3	10	545.41	0	0.96	0.87
2	14	552.89	7.47	0.02	0.87
4	9	553.94	8.52	0.01	0.87
1	20	562.45	17.03	0	0.87
5	7	569.49	24.08	0	0.86

*K* denotes for the number of estimable parameters, AICc for the second‐order variant of Akaike's information criterion, ΔAICc for AICc difference, ω for Akaike weight and *R* ^2^ for Nagelkerkes/Cragg & Uhlers pseudo‐*R* ^2^ of the models 1–5. Models are ordered from high to low weight.

**Table 4:** The logistic generalized linear model with the highest weight (ω = 0.96) describing the link between fish meal detection rate and detection time step (0–8 hr … 72–80 hr), meal size (large [350–540 g], medium [190–345 g] and small [15–170 g]), and share of a meal in the daily fish consumption (large [>79%] and small [<38%])

Predictor variable	Parameter estimate	*SE*	Lower 95% CI	Upper 95% CI	*z*‐value	*p*‐Value
*intercept*	−2.41	0.22	−2.85	−1.98	−10.83	<.001***
0–8 hr	−0.06	0.14	−0.33	0.20	−0.47	.64
8–24 hr	2.02	0.21	1.62	2.42	9.86	<.001***
24–32 hr	0.96	0.35	0.28	1.64	2.76	<.01**
32–48 hr	1.16	0.58	0.01	2.30	1.98	<.05*
48–56 hr	0.61	0.87	−1.09	2.32	0.70	.48
56–72 hr	0.51	1.23	−1.90	2.92	0.42	.68
Small meals	0.02	0.27	−0.51	0.55	0.07	.94
Medium meals	−0.49	0.16	−0.80	−0.17	−3.02	<.01**
Small meal share	−0.86	0.26	−1.38	−0.35	−3.30	<.001***

Large meal size and large meal share were used as base categories for dummy coding and are thus not displayed; forward difference coding was used for the variable time step; thus, coefficients can be interpreted as the difference between a respective time step and the following one.

Significances: *p < 0.05, **p < 0.01, ***p < 0.001

**Figure 4:**
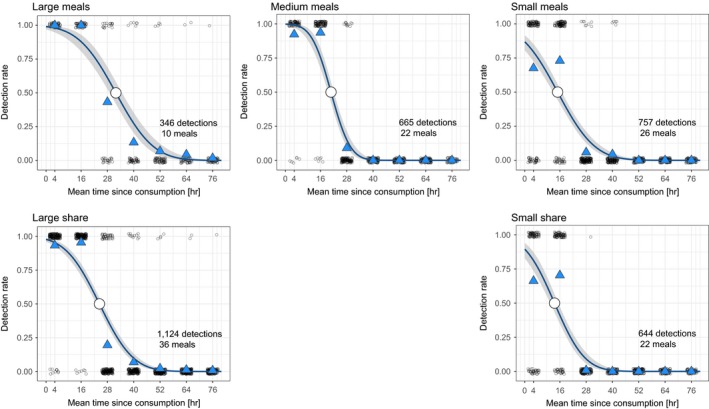
The PROBIT models for large (350–540 g), medium (190–345 g) and small (15–170 g) meals, as well as large (>79%) and small (<38%) per fish species shares of the daily consumption including their 95% confidence intervals. The mean of each detection interval is used as the *x*‐axis scale. All single detection events are displayed as bulked black circles. The number of detections is based on the number of meals times the fish species consumed in this size/share category times the number of faeces collected at the seven time steps. The mean detection rate per time interval (blue triangles), as well as the time of 50% detection probability (white circle; large meals: 31.85 hr, medium meals: 20.78 hr, small meals: 14.98 hr, large shares: 24.35 hr, small shares: 13.56 hr) are depicted as well

Regarding the analysis of detection as a binary variable (iii), the PROBIT regressions for all five categories (large/medium/small meal and large/small share) contained significantly negative coefficients for the time postfeeding (Table 5, Figure 4). For large meals, the time of 50% detection probability was at 31.85 hr postfeeding, which was significantly longer than for the other four categories (Figure 4). With the exception of small meals vs. small shares, the 95% CIs did not overlap for any of the other categories, indicating a significant difference in detection probability over time (Table 6). At 28 hr postfeeding, the detection probability of large meals was 3.59 and 4.07 times higher as compared to small and medium meals, respectively. Large shares of the daily consumption were 4.22 times more likely than small shares to be detected at this time point.

**Table 5:** The PROBIT models describing the link between detection as a binary dependent variable (yes/no) and time as a continuous variable separately for large (350–540 g; 346 yes/no events), medium (190–345 g; 665 yes/no events) and small (15–170 g; 757 yes/no events) meals as well as large (>79%; 1,124 yes/no events) and small (<38%; 644 yes/no events) meal shares

Data subset	*R* ^2^	Predictor variable	Parameter estimate	*SE*	Lower 95% CI	Upper 95% CI	*z*‐value	*p*‐Value
Large meals	0.76	*intercept*	2.35	0.24	1.87	2.83	9.59	<.001***
		time	–0.07	0.01	–0.09	–0.06	–10.91	<.001***
Medium meals	0.83	*intercept*	2.99	0.30	2.42	3.57	10.13	<.001***
		time	–0.14	0.01	–0.17	–0.12	–11.21	<.001***
Small meals	0.60	*intercept*	1.12	0.13	0.86	1.38	8.51	<.001***
		time	–0.07	0.01	–0.09	–0.06	–12.78	<.001***
Large meal share	0.74	*intercept*	2.01	0.13	1.75	2.27	15.15	<.001***
		time	–0.08	0.00	–0.09	–0.07	–17.73	<.001***
Small meal share	0.64	*intercept*	1.25	0.16	0.95	1.56	8.02	<.001***
		time	–0.09	0.01	–0.11	–0.08	–11.51	<.001***

*R* ^2^ denotes for Nagelkerkes/Cragg & Uhlers pseudo‐*R* ^2^.

Significances: ***p < 0.001

**Table 6:** The time of 50% detection probability post consumption including 95% confidence intervals (upper and lower CI) for the meal size and meal share categories

Data subset	50% detection probability (h)	Lower 95% CI	Upper 95% CI
Large meals	31.85	29.07	34.63
Medium meals	20.78	19.47	22.09
Small meals	14.98	13.05	16.91
Large meal share	24.35	22.95	25.75
Small meal share	13.56	11.76	15.37

## 4 DISCUSSION

The molecular approach allowed, as hypothesized in H1, the reliable detection of fish DNA in faeces and pellets of cormorants. Contrastingly, only two fish species could be identified via morphological analysis of indigestible prey remains in pellets. Prey detections happened from 1.5–2.5 hr up to 72–80 hr after the birds consumed fish. However, only 12% of the consumed fish meals were detected more than 32 hr postfeeding. Independent of fish species, large meal shares (>79% based on the daily consumption) had a significantly positive effect on per‐meal detection rates in faeces as compared to small meal shares. Small meals and meal shares were only detectable at the initial three samplings, and the time point of 50% detection probability was significantly longer postfeeding for large vs. medium and small meal sizes, as well as large vs. small meal shares, supporting hypothesis H2.

### 4.1 The effect of meal size and share on prey DNA detectability

In the PROBIT‐based analysis of faeces, large meals had a significantly higher per‐meal detection rate over time, in line with results of invertebrate feeding trials where a positive relationship between meal size and prey DNA detection success over time was observed (e.g., Carreon‐Martinez et al., 2011; King et al., 2010). Whilst small meals and small proportions of prey species (minimum 2.9%) have been detected in scat samples of Stellar sea lions (*Eumetopias jubatus*) and harbour seals (*Phoca vitulina*) (Bowles, Schulte, Tollit, Deagle, & Trites, 2011; Deagle et al., 2005, 2013), this was not generally the case for the present experiment. Small meals were reliably detected when looking at the overall, faeces‐based DNA detection rates in the cormorant feeding trial (pooled over birds and experimental runs). However, large meals had detection rates of 100% at the first two time steps, whilst small meals only reached an overall detection rate of 62% at 0–8 hr and 8–24 hr. A probable explanation for this discrepancy is that prey DNA is not evenly distributed in cormorant faeces as this has already been hypothesized for Stellar sea lions (Deagle et al., 2005). Furthermore, pinniped faeces were homogenized prior to sample processing, increasing the chance to detect prey, which was fed in small quantities (Bowles et al., 2011; Deagle et al., 2005, 2013). On the contrary, the DNA of a single small fish consumed by a cormorant, which defecates several times throughout the digestion of a meal, would be only detectable in a fraction of the total faecal material produced that day. Nevertheless, the logistic generalized linear model did only report a significant difference between medium and large meal size as well as between small and large meal shares with negative coefficients corresponding with a reduction of size and share. This was not the case for the comparison between large and small meal size: based on the analysed data set, no significant differences in the probabilities of reaching a certain detection rate (from 0 to 1) could be detected between large and small meals. As detections were summarized per sampling and meal for this analysis and only 10 of the 58 fish meals were of large meal size, the comparably small data set could well explain the lack of such a significant difference.

Within the group of small meals, a distinct difference between meals contributing a large or small share to the daily consumption was observed: DNA of the former was detectable until 32–48 hr postfeeding, whilst detections of the latter happened with one exception only until 8–24 hr. The consumption of a small meal in combination with a large one might, as outlined above, have led to a faster evacuation of DNA from the digestive tract. This was not the case if the cormorant refrained from the consumption of the large meal. Moreover, the general pattern of large, medium and small meal detections observed in our study is similar to the results obtained for Herring Gulls (*Larus argentatus*) fed large, medium and small whiting (*Merlangius merlangus*) (Hilton, Houston, & Furness, 1998): medium and large meals produced higher amounts of faecal material at an initial peak and additionally, larger meal size led to slower declining defecation rates.

Regarding calculations of postfeeding prey detection probability over time, Greenstone et al. (2014) suggest constant meal sizes in feeding trials to facilitate the interpretation of field‐derived data. In the present study, these guidelines could not be applied for three reasons: first, the effect of changing meal sizes was the core focus of this work. Second, fish species were only available at certain sizes and their body mass varied to a certain extent. Third, large meals had to be offered as a composition of several individual fish (exception *S. fontinalis*), because cormorants otherwise refrained from consumption. The birds could not be force‐fed; thus, consumption of the entire daily ration could not be ensured inducing additional variation. Future trials investigating effects of meal size and meal share should definitely try to reduce variation by feeding a base diet and adding or replacing it by only one prey species, ideally with constant sizes for both species. Nevertheless, we minimized confounding effects of fragment length and primer efficacy in the presented trial and detection probability over time was calculated separately for the meal size and meal share categories independent of prey species after initial analysis revealed significant differences in prey DNA detection success between these groups. In contrast to other studies (Greenstone et al., 2014), the underlying data set was pooled across the whole experiment including four birds, three experimental runs and multiple faeces per bird and sampling tested for five prey species. The PROBIT models showed 50% detection probability to increase ~6 hr from small‐to‐medium meals, another ~11 hr from medium to large meals and ~10 hr from small to large shares (Table 3). Even though cormorant and experimental run were not found to improve the model fit, these results need to be interpreted with care because their effect could not be removed from further calculations. The limited number of samplings and the uneven distribution of sampling times due to short periods of daylight during the experiment (9.5–10.5 hr) also potentially influenced the presented results. Fortunately, the temperatures during the trial were constantly low (monthly mean 4.6°C) so even though samples were not frozen immediately after defecation, the degrading effect of temperature was kept at a minimum.

Previous studies investigating molecular prey detection in dietary samples of piscivores found methodological effects induced by different lengths of the amplified target DNA fragments (Deagle, Eveson, & Jarman, 2006) and additionally, an influence of prey lipid content on detectability in faeces (Thomas, Jarman, Haman, Trites, & Deagle, 2014). The herewith applied singleplex PCRs amplify fragments between 82 and 92 bp, thus minimizing potential fragment length effects. Furthermore, we evaluated potential confounding effects of amplification bias between different primer pairs by assessing primer efficacy through dilution series of target DNA templates. For all primer pairs, 10 target DNA ds present in PCR was the lowest dilution step for which reliable positive results (≥ 0.1 RFU) were obtained, indicating equal primer efficiency between primer pairs. Regarding the lipid content of consumed fish, the results of this experiment do not point at fish species as being a significant influence on prey DNA detection: *Coregonus* spp., a species with medium lipid content compared to the others included in the trial (Schreckenbach, Knosche, & Ebert, 2001) and consumed in the highest amounts, was detectable for the longest time postfeeding. Still, we cannot completely rule out such an effect, which might be masked by the large sampling interval and mass differences between individual meals.

### 4.2 Cormorant digestion physiology and molecular vs. morphological prey identification

The current results obtained by morphological analysis of prey remains concur with a series of previous feeding trials on cormorants (reviewed by McKay, Robinson, Carss, & Parrott, 2003). In these experiments, depending on the consumed fish species, pellets were not produced every day (Brugger, 1993; Cherubini & Mantovani, 1997; McKay et al., 2003) and generally contained the hard parts of fish consumed the previous day (Zijlstra & van Eerden, 1995). Additionally, hard part recovery rates in the present trial were highly prey‐specific with small fish more likely to be entirely digested, also confirming previous results (Brugger, 1993; McKay et al., 2003; Zijlstra & van Eerden, 1995). The benefits of molecular methods for investigations of trophic interactions have so far been demonstrated in a range of feeding trials on vertebrates (e.g., Bowles et al., 2011; Casper et al., 2007; Deagle et al., 2010; Egeter et al., 2015; Peters et al., 2015). This study is the first to apply molecular prey detection methods successfully to the faeces and pellets of cormorants within the context of a feeding trial. All fish species could be molecularly detected in pellets and always stemmed from the meal, which had been consumed the day before.

Previous nonmolecular studies on captive cormorants investigated digestive wear of fish hard parts, energy requirements and factors affecting gut retention time (Brugger, 1993; Gremillet, Storch, & Peters, 2000; Hilton et al., 1998; Zijlstra & van Eerden, 1995). Brugger (1993) found the minimum and maximum gut passage time of carmine dye, discarded with the liquid phase (uric acid) of cormorant faeces, to be <2 hr and >48 hr postfeeding, respectively. In this study, first prey detections happened in concurrence with previous results (Brugger, 1993) at 1.5–2.5 hr postfeeding but are based on the mean of a 2‐hr consumption time frame. This could potentially bias minimum gut passage time 1 hr up or down. White/grey faecal samples collected during the present feeding trial and consisting mainly of uric acid tested significantly less often positive for prey DNA, which in turn indicates that DNA surviving digestion is present in the “solid brown” part of cormorant excrements. Whilst the results of Jedlicka, Sharma, and Almeida (2013) prove inhibition through potentially co‐extracted inhibitory substances (e.g., bile salts) to be an issue regarding prey DNA extraction from bird faeces (Deuter, Peitsch, Hertel, & Muller, 1995; Kohn & Wayne, 1997; Zarzoso‐Lacoste, Corse, & Vidal, 2013), this was not the case in the presented study as spike PCRs showed no obstruction of DNA amplification.

After 24–32 hr postfeeding, faeces‐based prey DNA detections were scarce in the present experiment. However, 10% of the consumed meals were detectable beyond this point for up to 72–80 hr. In comparison, carmine dye fed to cormorants was detectable >48 hr and prey DNA was found up to 4 days postfeeding in the faeces of little penguins (Brugger, 1993; Deagle et al., 2010). The limited prey DNA detection probability beyond 24–32 hr can be attributed to the effect of the subsequent meals (“chaser prey”) (Greenstone et al., 2014). In vertebrate feeding trials, the test animals are usually not starved, but are offered ad libitum access to other food before and after the consumption of the target meal (Egeter et al., 2015), fed a daily ration with constant shares of different prey species (Casper et al., 2007; Deagle et al., 2010), or offered a different prey species (combination) every day (Thalinger et al., 2016). Although it is reasonable to assume nonstarving conditions for highly efficient hunters such as cormorants (Gremillet et al., 2001) with a calculated daily food intake of ~20% of their body mass (Zijlstra & van Eerden, 1995), the effect of dietary mixing and chaser prey has been controversially discussed for invertebrate feeding trials (Greenstone et al., 2014). There, the use of chaser prey sometimes leads to shorter detection intervals of the target meal (Weber & Lundgren, 2009). In the present study, the daily consumption of new fish species could have shortened the time span until last prey detection, as all remains of previous meals were forced to pass through the simple‐structured cormorant digestive tract by the following meals (Brugger, 1993; Hilton, Furness, & Houston, 2000). As the cormorants were not used to individual housing and sampling procedures, stress could have been another factor shortening gut passage times (Enck, Merlin, Erckenbrecht, & Wienbeck, 1989).

Finally, feeding trials involving vertebrates often have limitations concerning the number of participating animals and the time between sampling sessions: for example, Casper et al. (2007) checked the seal enclosure housing every 2 hr for fresh faeces, but only during daytime, whilst Egeter et al. (2015) collected faecal samples of *Rattus norvegicus*,* R. rattus*,* Mus musculus* and *Erinaceus europaeus* (*n* = 51) at 6‐hr intervals. The cormorants participating in the presented study were individually caged for 27 days and to keep stress levels in this isolated condition at a bearable level, samples could only be collected twice a day leading to smaller overall sample numbers and lower temporal resolution, which in turn could both lead to blurred gut retention times.

### 4.3 Implications for field‐collected dietary samples

Based on the herewith presented results, the following conclusions can be drawn for the interpretation of molecularly analysed field‐collected faeces and regurgitated pellets of cormorants: in faeces, detected prey DNA is substantially more likely to stem from a fish meal ≥170 g which has been consumed within the last 24 hr, whilst small meals ≤170 g have a maximum detection probability of ~85% right after gut passage if meals are consumed by cormorants in the field in similar proportions to this experiment. Consequently, large numbers of faeces are necessary to reliably detect small meals or rarely consumed small prey species in field‐collected samples (cf. Oehm, Thalinger, Eisenkolbl, & Traugott, 2017). Generally, meal size, meal share and sample collection time complicate the interpretation of DNA‐based data obtained from field‐collected faeces. Depending on the expected satiation of the studied piscivores (i.e., are they able to catch enough prey at the time and place of investigation), a small meal is under satiated conditions only detectable for a short time span post consumption. However, if the animal was only able to consume a small meal and further prey items are not accessible, a small meal (constituting a large share of the daily consumption) is detectable for a longer time. The use of regurgitated pellets for studies of the trophic ecology of cormorants circumvents these limitations by their daily production at dusk and by containing DNA and hard parts of prey fish consumed on the previous day.

## DATA ACCESSIBILITY

Data on the consumed fish as well as molecularly and morphologically obtained results of the feeding trial have been archived in the Dryad Digital Repository (https://doi.org/10.5061/dryad.cr922).

## AUTHOR CONTRIBUTIONS

B.T., J.O. and M.T. conceived and designed the study. B.T. and A.O. carried out the feeding trial and the molecular analysis. J.O. and A.O. carried out the morphological analysis. B.T. analysed the data, compiled tables and figures and wrote the manuscript, which was revised and improved by J.O. and M.T.
