# Supplementary material for: The influence of meal size on prey DNA detectability in piscivorous birds
Source: Mol Ecol Resour. 2017 Oct 14;17(6):e174–86. doi: 10.1111/1755-0998.12706 (PMC5725817; doi:10.1111/1755-0998.12706)
Supplement: Supplementary file 1 [file MEN-17-e174-s001.docx]

**The influence of meal size on prey DNA detectability in piscivorous birds**

Bettina Thalinger, Johannes Oehm, Armin Obwexer & Michael Traugott

*Institute of Ecology, University of Innsbruck, Technikerstr. 25, 6020 Innsbruck, Austria*

**SI 1: Molecular sexing of captive cormorants**

For lysis of the feather samples, the *inferior umbilicus* was cut off and placed in a 1.5 ml reaction tube. After adding 100 µl lysis buffer, the “feather tips” were incubated on a rocking platform over night at 56 °C. DNA extraction was carried out as for all other sample types in the presented manuscript, except feather-samples were eluted in only 100 µl TE-buffer.

The feather extracts were subjected to molecular sexing via PCR using the primers 2550F and 2718R, targeting the chromodomain-helicase-DNA-binding protein 1 (CHD1) gene (Fridolfsson and Ellegren, 1999). Each 10 µl reaction contained 0.25 U OneTaq® DNA polymerase (NEB, Ipswich, USA), 1 × reaction buffer (NEB) and additional MgCl_2_ to a final concentration of 3 mM, 0.2 mM dNTPs (Genecraft, Köln, Germany), 5 μg bovine serum albumin (BSA), 0.5 μM of each primer, 1.5 μl of DNA extract, and PCR-grade water to adjust the volume. The following thermocycling conditions were used for amplification: 2 min at 94 °C, 35 cycles of 20 s at 94 °C, 30 s at 50 °C, 1 min at 68 °C, and once 3 min at 68 °C. For PCR product separation and analysis, the automatic capillary electrophoresis system QIAxcel and the QIAxcel Biocalculator software, version 3.2 (QIAxcel DNA screening kit, Method AL320, QIAGEN), were used. The detection threshold was set at 0.1 relative fluorescence units (RFU).

**References**

Fridolfsson, A. K. & Ellegren, H. 1999. A simple and universal method for molecular sexing of non-ratite birds. Journal of Avian Biology, 30: 116-121.
